# Intraorbital haematoma during a commercial flight: a case report

**DOI:** 10.1186/s12886-015-0034-y

**Published:** 2015-05-07

**Authors:** Alper Mete, Can Pamukcu, Ahmet Mete, Sabit Kimyon, Duçem Mete, İbrahim Gözen, Oğuzhan Saygılı

**Affiliations:** Department of Ophthalmology, Gaziantep University, School of Medicine, Gaziantep, 27310 Turkey; Department of Ophthalmology, Hatem Private Hospital, Gaziantep, Turkey; Department of Radiology, Gaziantep University, School of Medicine, Gaziantep, Turkey; Department of Ophthalmology, Sehitkamil Public Hospital, Gaziantep, Turkey; Department of Ophthalmology, Primer Private Hospital, Gaziantep, Turkey; Department of Radiology, Sehitkamil Public Hospital, Gaziantep, Turkey

**Keywords:** Intraorbital haematoma, Commercial flight, Subperiostal haematoma

## Abstract

**Background:**

Intraorbital haematoma is a rare clinical entity which can be caused by orbital traumas, neoplasms, surgeries nearby sinuses and orbit, vascular malformations, acute sinusitis, systemic abnormalities, barotrauma and valsalva maneuver.

**Case Presentation:**

A 74-year-old male presented with sudden onset of ocular pain, upper eye lid swelling, proptosis and diplopia after a commercial flight. After complete ophthalmic ocular examination including pupillary light reflexes and laboratory examinations; computed tomography and magnetic resonance imaging of orbit revealed a subperiostal mass-like lesion in the right retrobulbar-extraconal region which was compatible with intraorbital haematoma. Visual acuity was not compromised so we planned a conservative approach with close observation. We administered systemic corticosteroid and topical dorzolamide/timolol combination therapy. At the first month follow-up, intraorbital haematoma resolved without significant sequelae.

**Conclusion:**

Intraorbital haematoma can be managed by conservative approach without any intervention if it does not threat visual acuity or optic nerve. We experienced a case of intraorbital haematoma during a commercial flight. We discussed the rarity of this condition and its management.

## Background

Intraorbital haematoma is an uncommon entity in ophthalmology practice. It is mostly associated with orbital traumas, surgeries nearby sinuses and orbit, neoplasms, increased venous pressure, vascular malformations, acute sinusitis, bleeding disorders such as haemophilia and barotrauma [1,2]. Patients usually present with sudden onset of retroorbital pain, proptosis, acute visual loss and diplopia. Spontaneous intraorbital haematoma is poorly represented in existing literature, consisting of a few cases [3,4].

We experienced a case of intraorbital haematoma which caused painful swelling in the right upper eyelid, proptosis and diplopia to a passenger during a commercial flight. We discussed the rarity of this condition and its management.

## Case presentation

**Figure 1:**
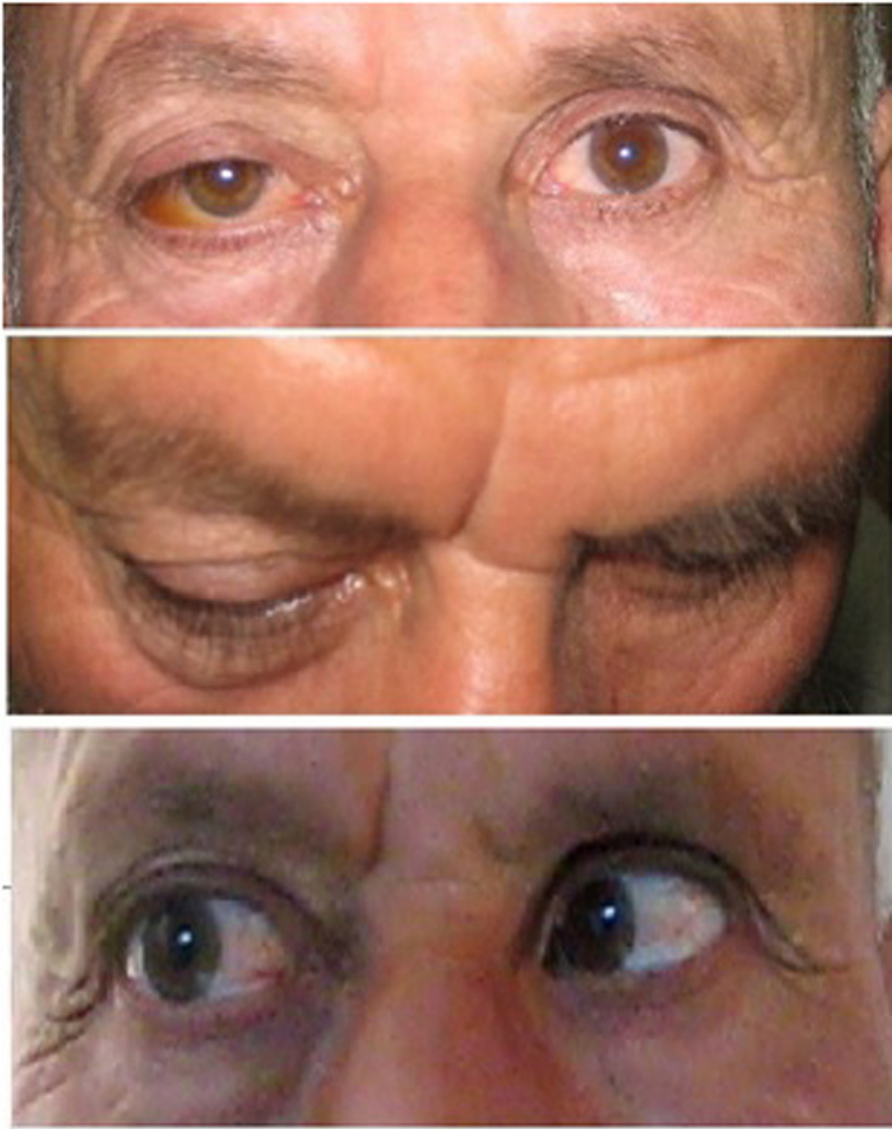
Patient had subconjunctival haemorrhage, proptosis and lateral gaze restriction in the right eye at presentation.

A 74-year-old male patient who was a passenger in Sydney to Istanbul commercial flight presented to our hospital with a history of sudden onset of painful swelling in the right eye which occurred 8 hours ago, while the aircraft was descending for landing. The patient had subconjunctival haemorrhage, upper eyelid swelling and proptosis in the right eye. We performed a complete ophthalmic examination. Best corrected visual acuity was 20/20 in both eyes. Direct and indirect pupillary light reflexes were normal. We didn’t observe relative afferent pupillary defect. There wasn’t any colour vision deficiency. The Hertel exophthalmometry measurements were 19 mm and 17 mm in the right and left eye respectively. There was lateral gaze restriction in the right eye which was causing diplopia (Figure 1). Intraocular pressure (IOP) was 21 mmHg in right eye and 16 mmHg in left eye which was measured with Goldmann applanation tonometry. Dilated fundus examination was normal.

**Figure 2:**
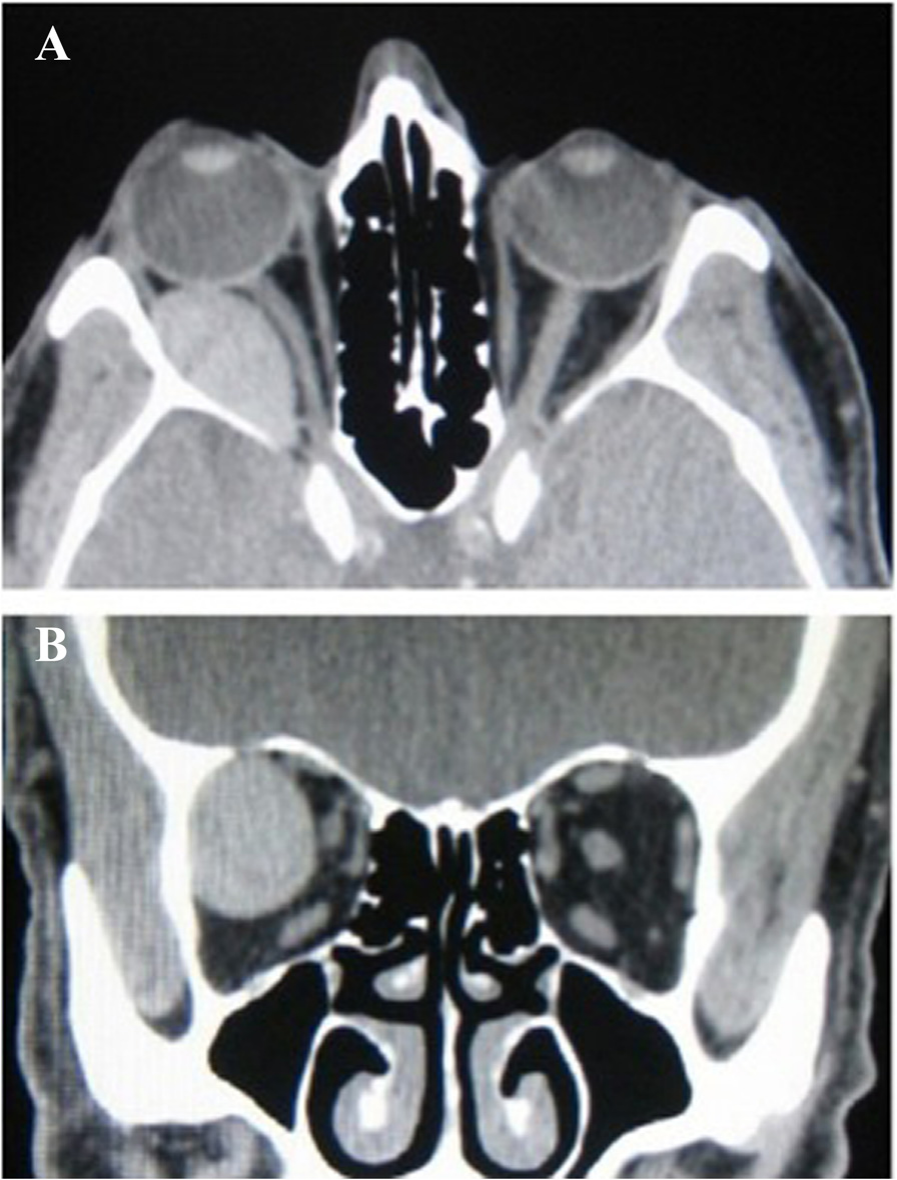
Axial **(A)** and Coronal **(B)** CT images of orbit revealed high density mass-like lesion in retrobulbar-extraconal part of right orbit consistent with subperiostal haematoma which displaces bulbus oculi, lateral rectus muscle and optic nerve.

**Figure 3:**
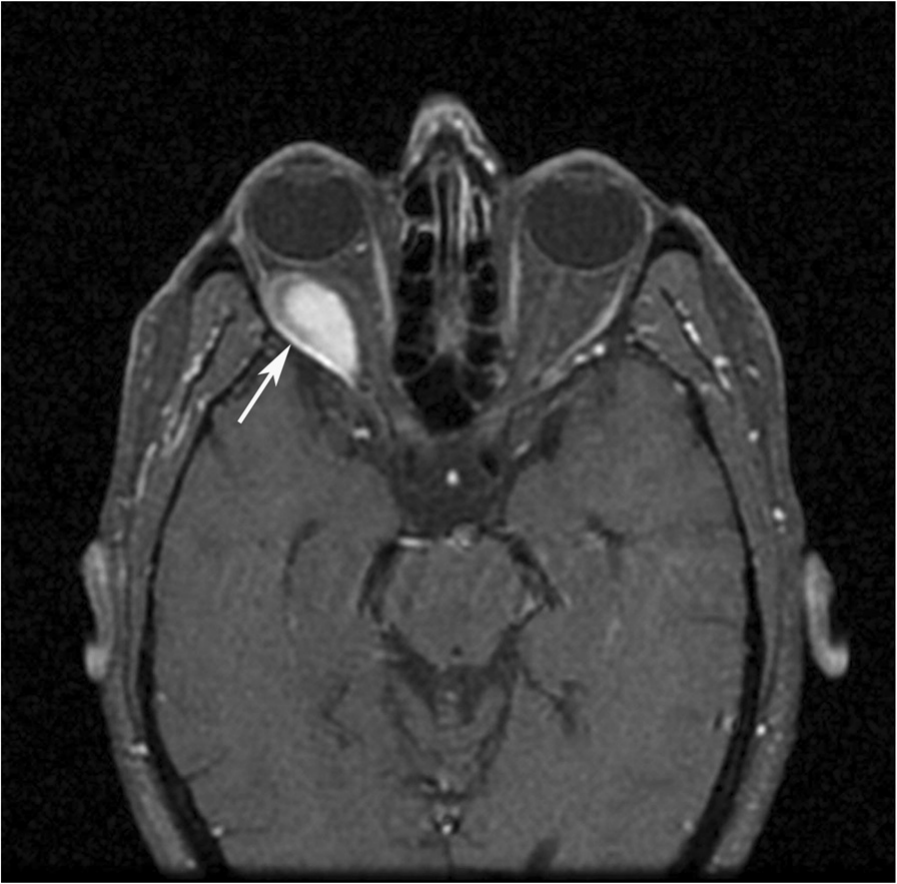
Axial fat sat T1-weighted MRI which showed ovoid shaped hyperintensity at right retrobulbar-extraconal region consistent with subperiostal haematoma.

There weren’t any systemic disease, medication, bleeding disorder, surgery or general anaesthesia, sinus infection or mucocele and previous trauma in the medical history of the patient. Laboratory examinations were within normal limits. Computed tomography (CT) revealed a subperisotal haematoma at the retrobulbar-extraconal region of the right orbit (Figure 2). We did an orbital magnetic resonance imaging (MRI) to support our diagnosis and the results confirmed haematoma in the right orbit (Figure 3).

**Figure 4:**
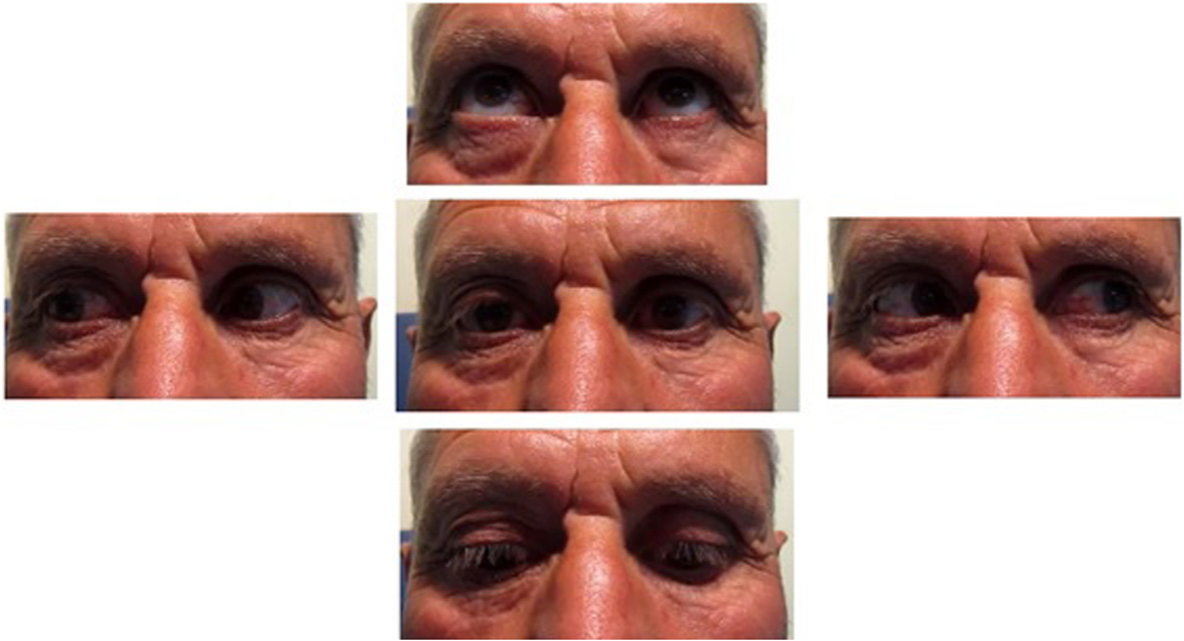
Proptosis and lateral gaze restriction recovered at the first month follow-up. Ocular movements were normal in all directions.

We adopted a conservative approach with close observation. Systemic corticosteroid and topical dorzolamide/timolol combination were administered to the right eye of the patient. We followed up visual acuity, pupillary reflexes, IOP, Hertel exophthalmometry and fundus examinations every 4 hours for the first two days and then daily for two weeks. After 24 hours, IOP decreased to 18 mmHg; visual acuity, pupillary reflexes and fundus examination were normal. The patient was stable for the following 10 days. At the 19^th^ day, patient had minimal subconjonctival haemorrhage and slight proptosis whereas upper eyelid swelling, lateral gaze restriction and diplopia resolved. We stopped topical dorzolamide/timolol combination and reduced systemic corticosteroid dosage. Intraorbital haematoma resolved without sequelae on the control CT at the first month follow-up (Figure 4) so that we stopped all medication. At the third month follow-up, patient didn’t have any sequelae and complete ophthalmic examination was normal.

## Discussion

Intraorbital haematoma is an uncommon clinical entity in healthy individuals which is generally caused by surgery or trauma around the orbit, vascular malformations, increased venous pressure, valsalva maneuver, bleeding disorders, infection, inflammation, neoplastic or non-neoplastic orbital lesions and sinusitis. It may also occur secondary to intense paroxysmal cough, valsalva maneuver, during sickle cell crisis, general anaesthesia and barotrauma [1-5]. The symptoms are; sudden onset of retroorbital pain, proptosis, acute visual loss, chemosis and diplopia which occurs abruptly or gradually [6,7]. The diagnosis is confirmed by CT, MRI and laboratory examinations. Haematoma is hyperdense at early periods and its hyperdensity decreases at late periods in CT while MRI shows hyperintensity in T2 images and intermediate signal intensity on T1 images at hyperacute period. T1 and T2 images become hypointense at chronic period when the haematoma liquefies. Cerebral angiography is also useful to diagnose vascular anomalies, if needed. [1,5,6,8].

Airline travel is safe and reasonably comfortable but many factors including psychological stress, jet lag, and pre-existing diseases can affect a small number of passengers to become sick [9,10]. The etiology of the haemorrhage in this case can be explained by two hypotheses. First of all; psychological stress might have caused a systemic hypertension or valsalva maneuver which led to an increase in systemic and orbital venous pressure [11]. Secondly; sudden changes in cabin pressure might have caused barotrauma. Several cases of intraorbital haematoma have been reported in scuba divers suffering barotrauma [12-15]. This form of barotrauma is not from a sudden increase in cranial and orbital venous pressure, but rather a fall in pressure in the diver’s mask leading to a suction effect on the orbital contents and an increase in the pressure differential between the veins and the orbital soft tissues. In the history of the patient there was sudden onset of painful swelling in the right eye while aircraft was descending for landing. Diving barotrauma usually occurs during ascent because of a decrease in diver’s mask pressure whereas haematoma occurred during descent in this case, so we thought that increased orbital venous pressure caused by a valsalva maneuver is a more likely etiology for our patient.

If the clinical presentation and imaging features are consistent with a diagnosis of intraorbital haemorrhage and the vision is not compromised then a conservative approach can be adopted because in many patients blood resorbs without sequelae. Corticosteroids are the first line of treatment in intraorbital haemorrhages [1]. If the patient has visual loss or optic nerve involvement which reveals itself with afferent pupillary defect or dyschromatopsia, orbital decompression surgery or needle aspiration should be the first choice of treatment [7,16]. Intraorbital haemorrhage cases may require surgical or endoscopic decompression if the mass effect persists in spite of medical treatment [1,7]. Drainage of haematoma can be considered if the vision is compromised, if there is relative afferent pupillary defect or if proptosis is severe with the risk of corneal exposure. This can be performed in cases with spontaneous orbital haematoma, orbital haematoma associated with extraocular muscles or orbital floor implants. Haematoma should be drained and implant should be removed to avoid recurrence of bleeding with orbital floor implants [1,7-17]. Our patient’s clinical presentation and imaging features were consistent with a diagnosis of limited subperiostal intraorbital haematoma. We observed mild increase in IOP, proptosis and diplopia. Visual acuity was not compromised. We adopted conservative approach with close observation because haematomas without any visual disturbance have good prognosis and usually resolve without significant sequelae in a week or a month [1,7].

## Conclusion

Intraorbital haematoma can be managed by conservative approach without any interventions if it does not threat visual acuity or optic nerve. Intraorbital haematomas have good prognosis and usually resolve without significant sequelae. Psychological stress may cause a systemic hypertension or valsalva maneuver which leads to an increase in systemic and orbital venous pressure during flights and to our knowledge this is the first case presented with intraorbital subperiostal haematoma during a commercial flight.

### Consent

Written informed consent was obtained from the patient for publication of this case report and any accompanying images. A copy of the written consent is available for review by the Editor of this journal.

## Abbreviations

CT: Computed Tomography
MRI: Magnetic Resonance Imaging
IOP: Intraocular Pressure
